# Hrg1 promotes heme-iron recycling during hemolysis in the zebrafish kidney

**DOI:** 10.1371/journal.pgen.1007665

**Published:** 2018-09-24

**Authors:** Jianbing Zhang, Ian Chambers, Sijung Yun, John Phillips, Michael Krause, Iqbal Hamza

**Affiliations:** 1 Department of Animal & Avian Sciences and Department of Cell Biology & Molecular Genetics, University of Maryland, Maryland, United States of America; 2 Laboratory of Molecular Biology, National Institute of Diabetes and Digestive and Kidney, Diseases, National Institutes of Health, Bethesda, Maryland, United States of America; 3 Division of Hematology, University of Utah School of Medicine, Salt Lake City, Utah, United States of America; Marine Biological Laboratory, UNITED STATES

## Abstract

Heme-iron recycling from senescent red blood cells (erythrophagocytosis) accounts for the majority of total body iron in humans. Studies in cultured cells have ascribed a role for HRG1/SLC48A1 in heme-iron transport but the *in vivo* function of this heme transporter is unclear. Here we present genetic evidence in a zebrafish model that Hrg1 is essential for macrophage-mediated heme-iron recycling during erythrophagocytosis in the kidney. Furthermore, we show that zebrafish Hrg1a and its paralog Hrg1b are functional heme transporters, and genetic ablation of both transporters in double knockout (*DKO)* animals shows lower iron accumulation concomitant with higher amounts of heme sequestered in kidney macrophages. RNA-seq analyses of DKO kidney revealed large-scale perturbation in genes related to heme, iron metabolism and immune functions. Taken together, our results establish the kidney as the major organ for erythrophagocytosis and identify Hrg1 as an important regulator of heme-iron recycling by macrophages in the adult zebrafish.

## Author summary

Total body iron stores in mammals is a composite of iron absorption from diet and iron recycled by macrophages from dying red blood cells (RBCs). Upon erythrophagocytosis of RBCs, the hemoglobin is degraded and heme is imported from the phagosomal compartment into the cytoplasm so that the iron can be released from heme. Defects in these pathways can lead to aberrant iron homeostasis. The Heme Responsive Gene-1 (HRG1, SLC48A1) was identified previously as a heme importer in the intestine of the roundworm, *Caenorhabditis elegans*. In cell culture studies, HRG1 was demonstrated to mobilize heme from the erythrophagosome of mouse macrophages into the cytosol. However, the *in vivo* function of HRG1 remains to be elucidated. The zebrafish is a powerful genetic animal model for studying vertebrate development and ontogeny of hematopoiesis. In zebrafish, the kidney marrow is the adult hematopoietic organ that is functionally analogous to the mammalian bone marrow. In this study, we show that Hrg1 plays an essential *in vivo* role in recycling of damaged RBCs, and that the kidney macrophages are primarily responsible for recycling heme-iron in the adult zebrafish.

## Introduction

Heme is an iron-containing porphyrin that acts as an essential cofactor in numerous biological processes such as oxygen transport, miRNA processing, electron transfer, and circadian clock control [1]. In humans, total body iron store is a composite of dietary iron absorption, which accounts for around 10% of the daily iron requirement after compensating for daily iron losses, and heme-iron recycling through clearance of senescent red blood cells (RBCs) [2, 3]. RBCs contain approximately 70% of the overall body iron in the form of heme, and consequently heme-iron recycling is a significant contributor of systemic iron homeostasis [4]. Thus, a better understanding of heme recycling and transport is critical to understanding the role of heme and iron in red cell synthesis and turnover.

The lifespan of mature RBCs is limited in circulation, with approximately40 and 120 days for mouse and human, respectively [5, 6]. Furthermore, circulating RBCs can be subjected to damage under stress conditions leading to hemolysis. When RBCs become senescent or the number of damaged RBCs increases in the circulation, macrophages from the reticuloendothelial system (RES, spleen and liver) contribute to RBC clearance and subsequently promote heme-iron recycling through erythrophagocytosis (EP). Upon degradation of RBCs in the erythrophagosome, heme is imported into the cytoplasm for degradation by the heme-degrading enzyme heme oxygenase-1 (HMOX1) [7]. Defects in erythrophagocytosis (EP) lead to aberrant iron homeostasis, culminating in iron deficient anemia (IDA) or iron overload [8, 9].

The Heme Responsive Gene-1 (HRG1, SLC48A1) was identified previously as a heme importer in the intestine of *Caenorhabditis elegans* [10]. In *ex vivo* cultured mouse bone marrow derived macrophages (BMDMs), HRG1 was recruited to the erythrophagosome membranes where it colocalizes with NRAMP1, an iron transporter found on the phagolysosomal membranes, surrounding ingested senescent RBCs [11, 12]. *HRG1* mRNA is upregulated during erythrophagocytosis (EP) in mouse bone marrow derived macrophages (BMDMs) and HRG1 protein is strongly expressed in macrophages of the reticuloendothelial system (RES) and specifically localizes to the phagolysosomal membranes [11]. Depletion of HRG1 by siRNA in bone marrow derived macrophages (BMDMs) causes defective heme transport from the phagolysosomal compartments and a failure to upregulate *HMOX1* mRNA, demonstrating that HRG1 must mobilize heme from the erythrophagosome into the cytosol. However, whether HRG1 functions similarly *in vivo* remains to be elucidated.

The teleost fish, *Danio rerio*(zebrafish), is a powerful genetic animal model for studying vertebrate development and ontogeny of hematopoiesis [13]. In zebrafish, the kidney marrow (head kidney) is the adult hematopoietic organ and is functionally analogous to the mammalian bone marrow. Compared to the kidney marrow, the zebrafish spleen is not well characterized, although it has been proposed to function as a reservoir for RBCs where erythrocytes are stored and destroyed [14]. Histological analysis reveals that macrophages, although rare in the zebrafish spleen, contain phagosomes with erythrocytes and other cellular debris, suggesting that erythrophagocytosis (EP) could occur in the zebrafish spleen [15, 16]. However, direct experimental evidence identifying the tissues that are involved in heme-iron recycling in the zebrafish is lacking. In this study, we show that Hrg1 plays an essential role in the recycling of damaged RBCs and that the kidney macrophages are primarily responsible for heme-iron recycling in the zebrafish.

## Results

### The zebrafish genome contains two *hrg1* paralogs

**Fig 1:**
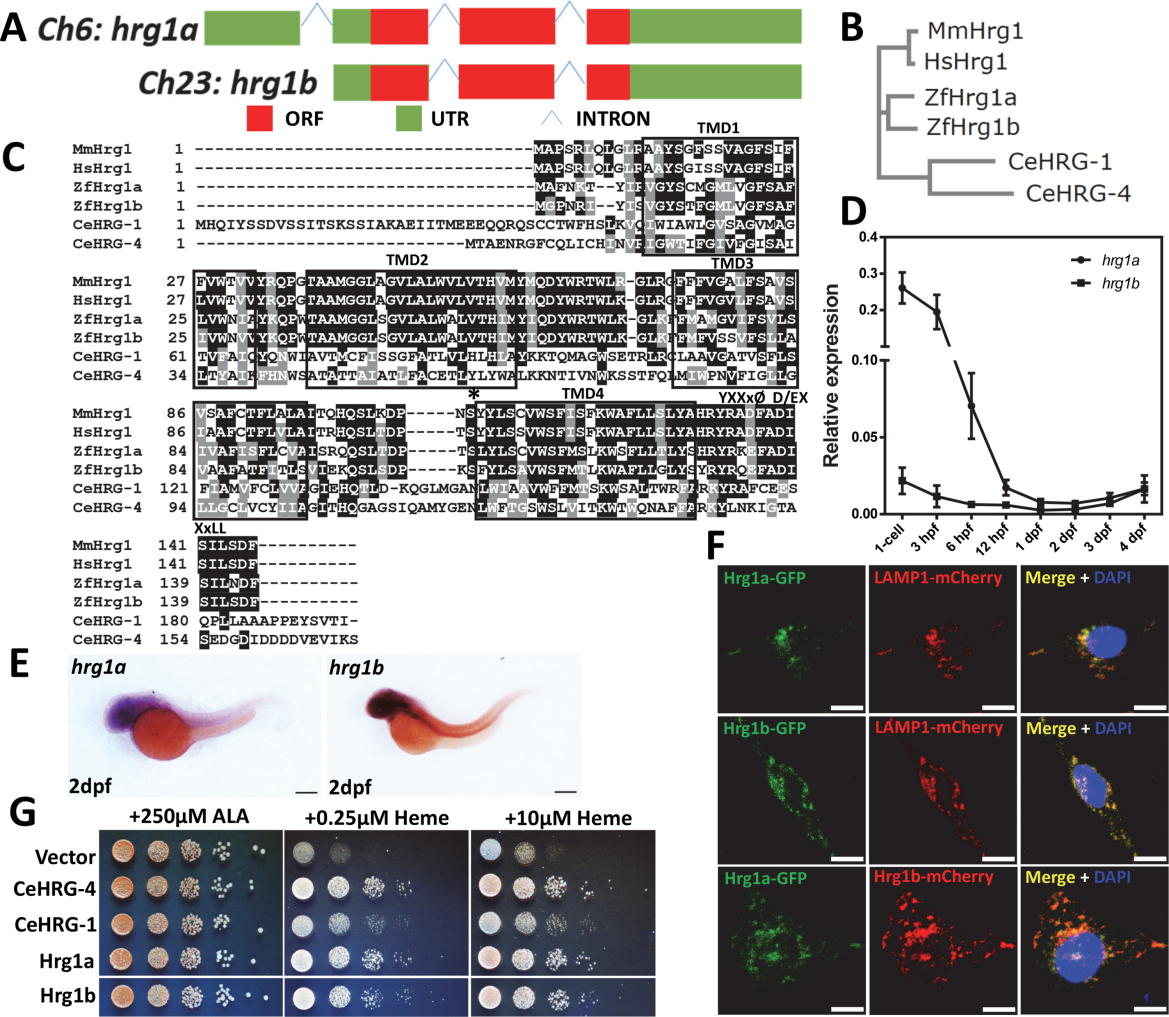
*Hrg1* is duplicated in the zebrafish genome. **(A)** Two *hrg1* homologs exist in zebrafish genome: *hrg1a* (slc48a1b) on chromosome 6 and *hrg1b* (slc48a1a) on chromosome 23. **(B)** Phylogenetic analysis of zebrafish Hrg1a and Hrg1b with CeHRG-1, CeHRG-4, MmHrg1, and Human Hrg1 (HsHrg1). Sequences were aligned using ClustalW and a phylogenetic tree was generated using the Neighbor-Joining method in MEGA5. **(C)** Multiple sequence alignment of zebrafish Hrg1a and Hrg1b with orthologs CeHRG-1, Cehrg-4, MmHrg1, and Human Hrg1 (HsHrg1). Asterisk, conserved histidine; black box, putative transmembrane domains; YXXxØ, C-terminal tyrosine sorting motif; D/EXXxLL, di-leucine sorting motif. **(D)** qRT-PCR of *hrg1a* and *hrg1b* in zebrafish embryos at different stages. 50 embryos before 24hpf were collected as one cohort. For embryos later than 24hpf, 30 were pooled as one cohort. 3 cohorts of each stage were harvested for total RNA extraction. Expression level was normalized to *ß-actin*. **(E)** Lateral view of *hrg1a* and *hrg1b* expression by WISH at 2dpf. Anterior is to the left. Scale bar: 200μm. **(F)** Confocal microscopy depicting subcellular localization of Hrg1a and Hrg1b with C-terminally tagged fluorescent proteins in transfected HEK-293 cells. Lamp1 is a marker for lysosomal compartments. Scale bar: 10 μm. **(G)** Yeast growth assay showing that zebrafish Hrg1a and Hrg1b are able to mediate heme transport in yeast. The *hem1Δ* strains transformed with empty vector pYes-DEST52, CeHRG-4, CeHRG-1, Hrg1a and Hrg1b were cultivated overnight and spotted in serial dilutions on SC plates supplemented with 250μM ALA and indicated concentrations of heme.

Owing to ancient whole-gene duplication in teleosts, the zebrafish genome can typically contain more than one orthologue per human protein-encoding gene [17, 18]. Indeed, two *hrg1* paralogs are present—*hrg1a* (*slc48a1b*) and *hrg1b* (*slc48a1a*) are located on chromosome 6 and chromosome 23, respectively (**Fig 1A**). Both paralogs are phylogenetically related to *C*. *elegans* (CeHRG-1 and CeHRG-4), mouse (MmHRG1) and human (HsHRG1) HRG1 (**Fig 1B and 1C**). Sequence alignment reveal that the protein sequences of Hrg1a and Hrg1b share 73% identity and 86% similarity to each other (**Fig 1C**). Protein topological predictions show that Hrg1a and Hrg1b contain four transmembrane domains with a cytoplasmic N- and C-terminus and conserved amino acids that have been implicated in heme transport (**Fig 1C**, asterisk) [19].

To determine the spatiotemporal expression of *hrg1*, total mRNA was extracted from embryos at different stages, from single cell to 4 days post fertilization (dpf). RT-PCR analysis revealed that the temporal expression patterns of *hrg1a* and *hrg1b* are similar and that both *hrg1a* and *hrg1b* mRNAs are present in the one-cell embryo (**Fig 1D, S1A Fig**). As zygotic mRNA expression is turned-on around 3 hour post fertilization, mRNA detected before this stage is typically associated with maternal deposition during oogenesis [20]. Whole-mount *in situ* hybridization (WISH) confirmed the RT-PCR analysis and revealed that *hrg1* is ubiquitously expressed throughout developing embryos, with high expression levels in the central nervous system **(Fig 1E, S1B and S1C Fig)**. qRT-PCR from dissected adult zebrafish tissues showed that *hrg1a* and *hrg1b* are expressed at different levels in various organs (**S1D and S1E Fig).**

Ectopic expression of fluorescent-tagged zebrafish Hrg1a and Hrg1b in HEK293 cells revealed that Hrg1a and Hrg1b colocalize with LAMP1, a lysosomal marker, consistent with previous studies that showed that the worm and human HRG1 localize to endo-lysosome-related organelles [10]. Moreover, coexpression of fluorescent-tagged Hrg1a and Hrg1b showed both proteins colocalize to similar intracellular compartments (**Fig 1F**). These results are consistent with the presence of potential heme-binding ligands and sorting motifs in the C-terminus of Hrg1a and Hrg1b (**Fig 1C**) [10, 11, 19]. Expression of zebrafish *hrg1a* and *hrg1b* rescued growth of *hem1Δ* yeast at heme concentrations as low as 0.25 μM, comparable to the established heme transporters CeHRG-4 and CeHRG-1, demonstrating that Hrg1a and Hrg1b are heme transporters (**Fig 1G**) [10].

### Zebrafish lacking Hrg1 show normal red cell development

To further define the function of zebrafish Hrg1 *in vivo*, we generated *hrg1a* and *hrg1b* double knockout zebrafish with CRISPR/Cas9 genome editing. The *hrg1a*^*iq261*^ mutant allele contains a 61 nt deletion and a 7 nt insertion (-61, +7) in exon 2, resulting in loss of its original ATG translation start site (**S2A Fig**). Although an ATG site downstream in the *hrg1a* ORF could be used for alternative translation initiation, this would cause a truncation of 38 amino acids at the N-terminus. The *hrg1b*^*iq361*^ mutant allele carries a -61 nt deletion in exon 3, resulting in a protein predicted to contain 28 less amino acids at the C-terminus and addition of 6 extra amino acids (**S2A Fig**).

**Fig 2:**
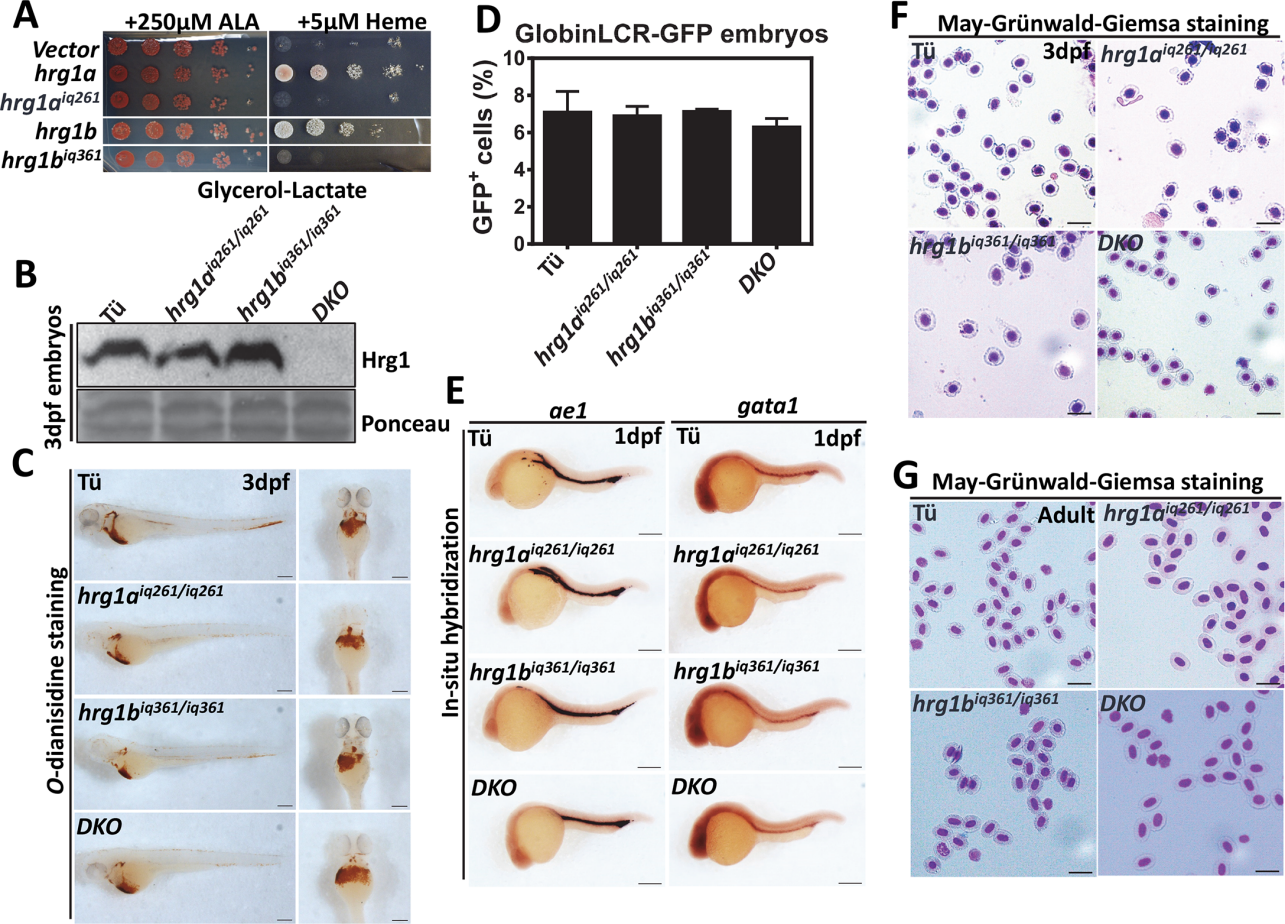
*hrg1 DKO* zebrafish survives to adulthood without overt hematological phenotypes. **(A)** Yeast growth assay showing that zebrafish Hrg1a^iq261^ and Hrg1b^iq361^ alleles are incapable of mediating heme transport in yeast in contrast to WT forms of Hrg1a and Hrg1b. The *hem1Δ* strain transformed with empty vector pYes-DEST52, *hrg1a*, *hrg1b*, predicted *hrg1a*^*iq261*^ and *hrg1b*^*iq361*^ were cultivated overnight and spotted in serial dilutions on SC plates supplemented with 250μM ALA and indicated concentration of heme. **(B)** Immunoblot of Hrg1 in membrane fractionation lysates from WT Tü, *hrg1a*^*iq261/iq261*^, *hrg1b*^*iq361/iq361*^ and double mutant *DKO*. Each crude membrane fraction lysate was from pooled ~30 embryos. Each lane represents 100 μg of protein from membrane fractionation lysates. Ponceau is used as a loading control for membrane fraction. **(C)** *O-*dianisidine staining of RBCs in 3dpf embryos from Tü, *hrg1a*^*iq261/iq261*^, *hrg1b*^*iq361/iq361*^ and *DKO* (n = 50 for each genotype). Scale bar: 200μm. **(D)** Quantitative percentages of GFP^+^ cells in Tü, *hrg1a*^*iq261/iq261*^, *hrg1b*^*iq361/iq361*^ and *DKO* mutants in GlobinLCR-GFP transgenic background. Error bars indicate SEM of 3 independent experiments (two-way ANOVA, p >0.05). **(E)** Lateral view of *ße1* and *gata1* expression by WISH at 1dpf embryos from Tü, *hrg1a*^*iq261/iq261*^, *hrg1b*^*iq361/iq361*^ and *DKO*. Anterior is to the left. N = 50 for each genotype. Scale bar: 200μm. **(F)** May-Grünwald-Giemsa staining of isolated peripheral RBCs at 3dpf embryos from Tü, *hrg1a*^*iq261/iq261*^, *hrg1b*^*iq361/iq361*^ and *hrg1a*^*iq261/iq261*^*; hrg1b*^*iq361/iq36*^ (N = 30). Scale bar: 20μm. **(G)** May-Grünwald-Giemsa staining of isolated peripheral RBCs from adult Tü, *hrg1a*^*iq261/iq261*^, *hrg1b*^*iq361/iq361*^ and *DKO*. Scale bar: 20μm.

To determine whether the truncated forms of Hrg1a and Hrg1b had residual function, we performed heme-dependent growth assays with the predicted mutant ORFs using the *hem1Δ* yeast mutant. Hrg1a^*iq261*^ and epitope-tagged Hrg1b^*iq361*^ mutant constructs were expressed, as determined by immunoblotting (**S2B Fig**), but failed to rescue *hem1Δ* growth even in the presence of 5 μM exogenous heme (**Fig 2A**). *hrg1* double knockout (*DKO*) zebrafish were generated by crossing *hrg1a*^*iq261*^ and *hrg1b*^*iq361*^ mutant alleles (**S2C Fig**). Intercrossing of *hrg1a*^*+/iq261*^*; hrg1b*^*+/iq361*^ generated progeny with the expected Mendelian ratios (**S2D Fig**) (chi-square test, *p > 0*.*05*) and survival rates for *DKO* fish were comparable to wildtype Tü and *hrg1a*^*iq261/iq261*^ or *hrg1b*^*iq361/iq36*^ single mutants. Immunoblotting of total membrane proteins obtained from 3 dpf embryos revealed no detectable Hrg1 protein in *DKO* fish (**Fig 2B**).

To evaluate whether *hrg1a*^*iq261/iq261*^, *hrg1b*^*iq361/iq361*^ or DKO mutants have defects in red cell homeostasis, we first evaluated red cell synthesis by collecting embryos and processing them for *o*-dianisidine staining which interrogates RBC hemoglobin production. No defects in embryonic hemoglobinization were detected at 3 dpf embryos (**Fig 2C**). Quantification of GFP-positive RBCs, in *hrg1* mutants crossed to the globinLCR-GFP transgenic fish, showed comparable numbers of RBCs demonstrating that neither globin expression nor RBC numbers were altered in the absence of *hrg1* (**Fig 2D, S2E Fig**) (Two-way ANOVA, *p>0*.*05*). Correspondingly, WISH showed unaltered expression of *gata1*, a transcription factor required for erythropoietic lineage specification, and *ae1*, a marker for developing RBCs, further indicating normal erythropoietic differentiation in *hrg1a*^*iq261/iq261*^, *hrg1b*^*iq361/iq361*^, and *DKO* mutant animals (**Fig 2E**) (n = 30). RBC morphology, as determined by May-Grünwald-Giemsa staining (**Fig 2F and 2G**), and hemoglobinization levels, as determined by *o-*dianisidine staining, were normal (**S2F and S2G Fig**) in RBCs from embryos and adults. Collectively, these results suggested that red cell differentiation and maturation in *hrg1a*^*iq261/iq261*^, *hrg1b*^*iq361/iq361*^, and *DKO* mutants were unaffected.

### Kidney macrophages are primary sites for red cell turnover in the zebrafish

**Fig 3:**
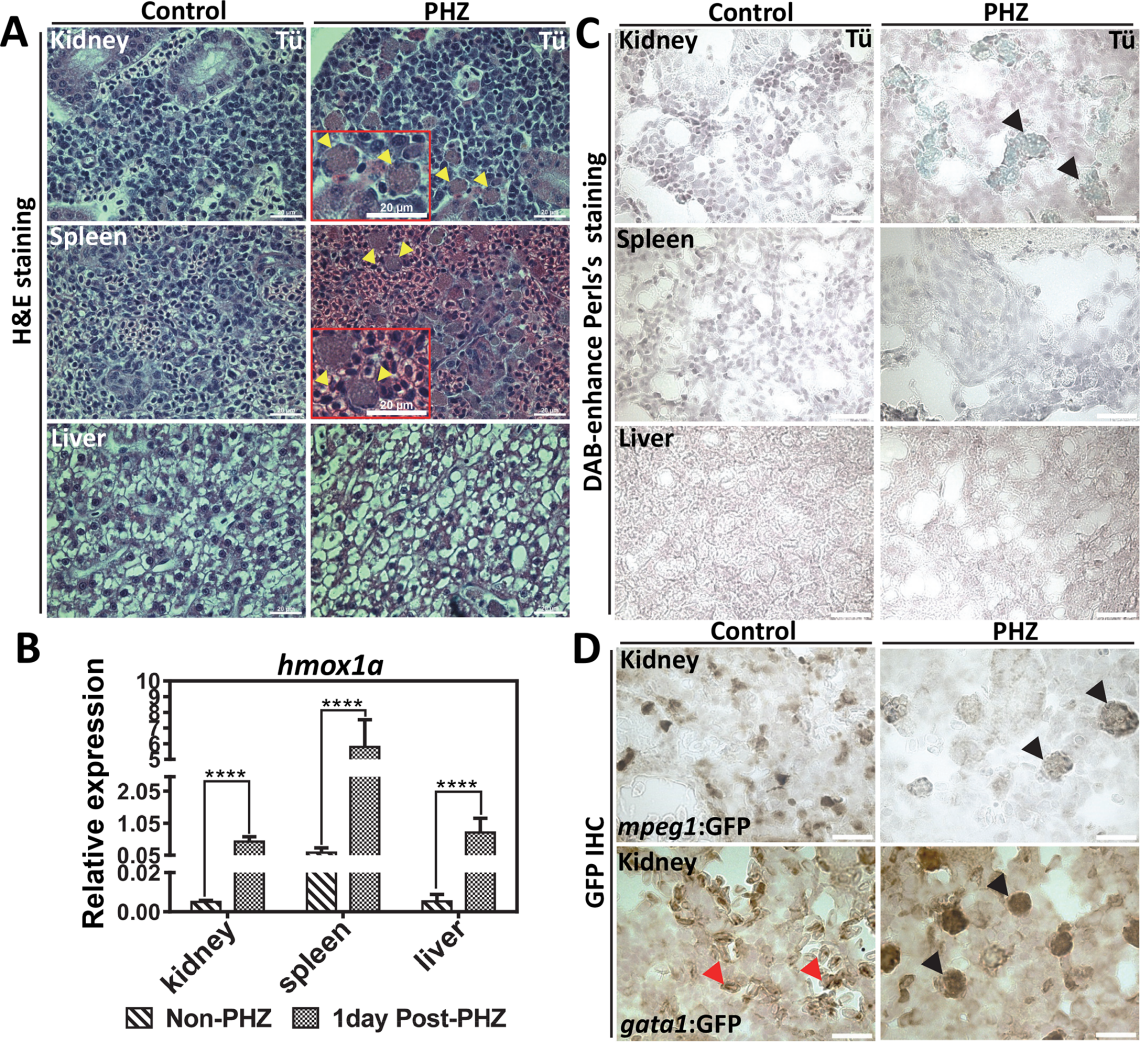
The zebrafish kidney is responsible for heme-iron recycling during EP. **(A)** H&E staining of kidney, spleen and liver sections from adult WT zebrafish with control (non-PHZ treated) and 1 day post PHZ-treatment samples. Cells undergoing erythrophagocytosis are indicated as yellow arrows. **(B)** qRT-PCR of *hmox1a* mRNA expression in the kidney, spleen and liver from control (non-PHZ treated) and PHZ treated adult zebrafish at 1day after treatment. Three adult zebrafish were pooled as one cohort, and three cohorts were repeated as biological triplicates. * p<0.05. **(C)** Perl’s Prussian blue iron staining on sections of kidney, spleen and liver from adult Tü WT zebrafish with control (non-PHZ treated) and 1 day post PHZ-treatment samples. Positive iron staining is indicated in kidney macrophages as showed by black arrows. **(D)** GFP IHC of kidney sections from adult transgenic zebrafish *gata1*:*gfp* and *mpeg1*:*gfp* with control (non-PHZ) and 1 day post PHZ-treatment. RBCs are indicated with red arrows and macrophages with black arrows.

Next, we determined whether Hrg1 plays a role in RBC degradation in the zebrafish. To analyze turnover, we had to first establish an *in vivo* model for hemolysis in the adult zebrafish and then locate the tissue involved in recycling damaged RBCs. To induce acute hemolysis and EP, phenylhydrazine (PHZ) was administrated to adult zebrafish. Histological analysis of the kidney, spleen, and liver conducted one day post-PHZ revealed infiltration of large-sized cells with irregular morphology in the kidney and spleen, but not the liver (**Fig 3A**, yellow arrows). qRT-PCR revealed that expression of the zebrafish *hmox1a* homolog was significantly upregulated in the kidney, spleen, and liver after PHZ treatment (**Fig 3B**). Perl’s Prussian blue showed iron-positive pigmentation only in the kidney macrophages but not in the spleen or liver (**Fig 3C**, yellow arrows), indicating that only the kidneys were responding to hemolysis for heme-iron recycling.

Since macrophage-specific antibodies are not available for the zebrafish, transgenic fish expressing *gata1*:*gfp* and *mpeg1*:*gfp*, which label the erythroid and macrophage cells respectively, were treated with PHZ to determine whether the large-sized cells were macrophages. Immunohistochemistry (IHC) with anti-GFP antibody detected GFP-positive cells in the kidney of PHZ-treated *mpeg1*:*gfp* zebrafish, indicating that the newly-populated cells are macrophages (**Fig 3D)**. Furthermore, PHZ-treatment of *gata1*:*gfp* fish enhanced GFP staining within macrophages while GFP staining was restricted only to erythroid cells in untreated fish (**Fig 3D**). By contrast, GFP staining was absent in the livers of transgenic and WT (Tü) zebrafish (**S3A and S3B Fig**). Together, these results suggest that macrophages populate the kidney to phagocytose RBCs in response to hemolysis, and that these cells are the primary sites for heme-iron recycling in the adult zebrafish.

### Zebrafish *hrg1* promotes heme-iron recycling in kidney macrophages

**Fig 4:**
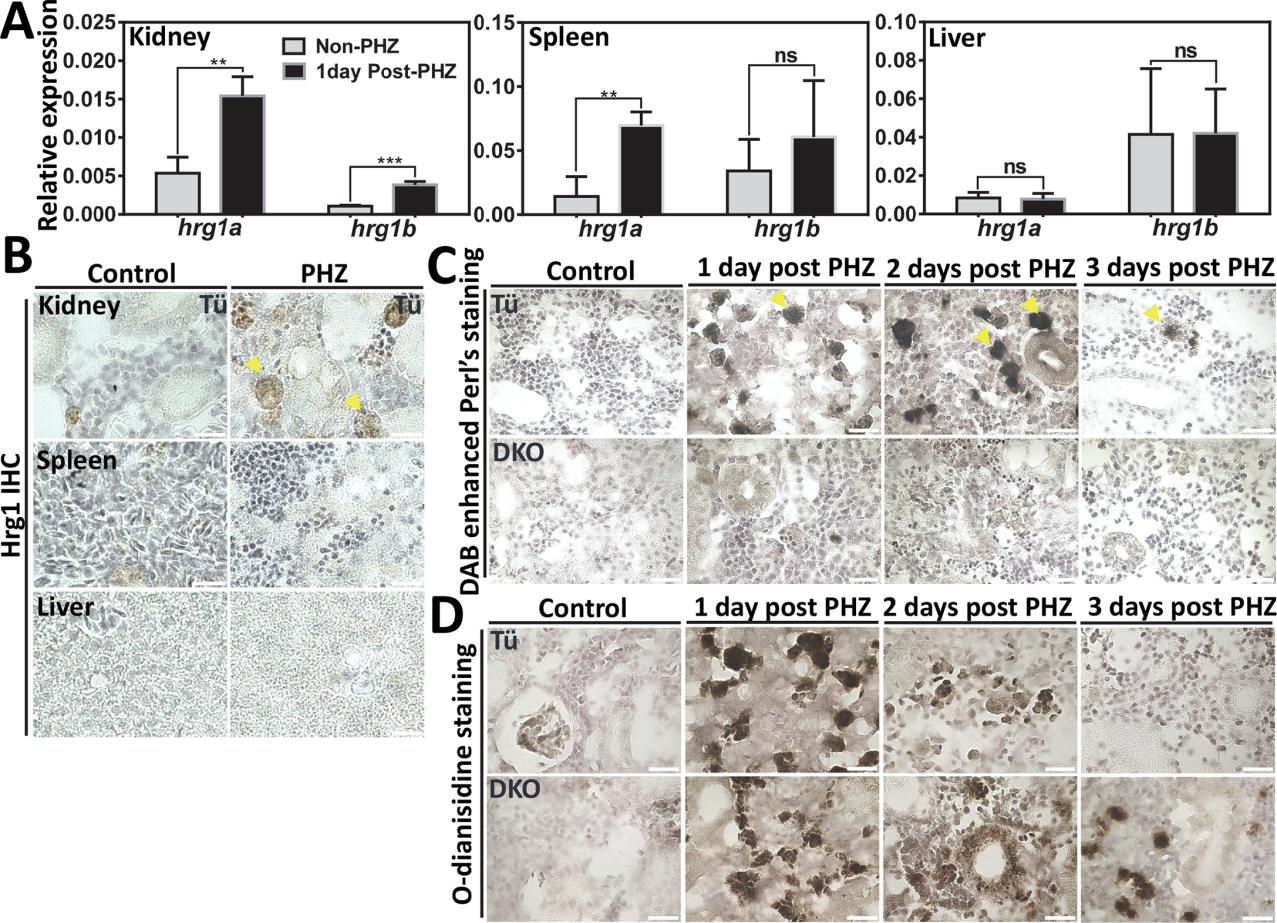
Defects of heme-iron recycling in kidney macrophages of Hrg1 DKO zebrafish. **(A)** qRT-PCR of *hrg1a* and *hrg1b* to quantify mRNA expression in the kidney, spleen and liver from control (non-PHZ treated) and 1-day post PHZ-treatment samples. Three adult zebrafish were pooled as one cohort, and three cohorts were repeated as biological triplicates. * p<0.05. **(B)** IHC staining of Hrg1 proteins in sections from the kidney, spleen and liver of adult zebrafish. **(C)** DAB-enhanced perl’s iron staining of kidney sections from Tü and *hrg1* DKO zebrafish with control (non-PHZ), 1 day, 2 days and 3 days post PHZ-treatment. **(D)** *O-*dianisidine staining of kidney sections from Tü and *hrg1* DKO zebrafish with control (non-PHZ), 1 day, 2 days and 3 days post PHZ-treatment. Scale bar: 20μm.

To assess whether *hrg1* plays a role in red cell turnover, we compared *hrg1* mRNA levels in tissues of adult zebrafish exposed to PHZ. qRT-PCR revealed that the levels of of *hrg1a* and *hrg1b* mRNA were significantly upregulated by PHZ in the kidney (**Fig 4A**). By contrast, only *hrg1a* was upregulated in the spleen, and neither were altered in the liver (**Fig 4A**). Consistent with these findings, IHC using anti-HRG1 antibodies showed increased Hrg1 staining after PHZ treatment in the kidney macrophages, while the signal was barely visible in the spleen and liver (**Fig 4B**).

DAB (3,3'-Diaminobenzidine)-enhanced Perl’s iron and Prussian blue staining detected iron accumulation in kidney macrophages of WT zebrafish at 1, 2 and 3 days post PHZ-treatment, but not in the kidneys of DKO zebrafish (**Fig 4D, S4A Fig**). In contrast to the WT kidneys, *o*-dianisidine staining revealed accumulation of heme in the kidneys of DKO zebrafish at 2 and 3 days after PHZ treatment (**Fig 4E**). No Perl’s iron staining above background could be detected in the spleens from WT or DKO zebrafish (**S4B Fig**). Importantly, the macrophage numbers or morphology in the kidney and spleen were unaltered in response to PHZ-induced hemolysis in the DKO mutants (**S4C and S4D Fig, yellow arrows**). These results further confirm that loss of Hrg1 results in the accumulation of heme in kidney macrophages due to defects in heme-iron recycling from damaged RBCs.

### Transcriptome analysis reveals that Hrg1 is an essential component of the heme and iron metabolism network in zebrafish

**Fig 5:**
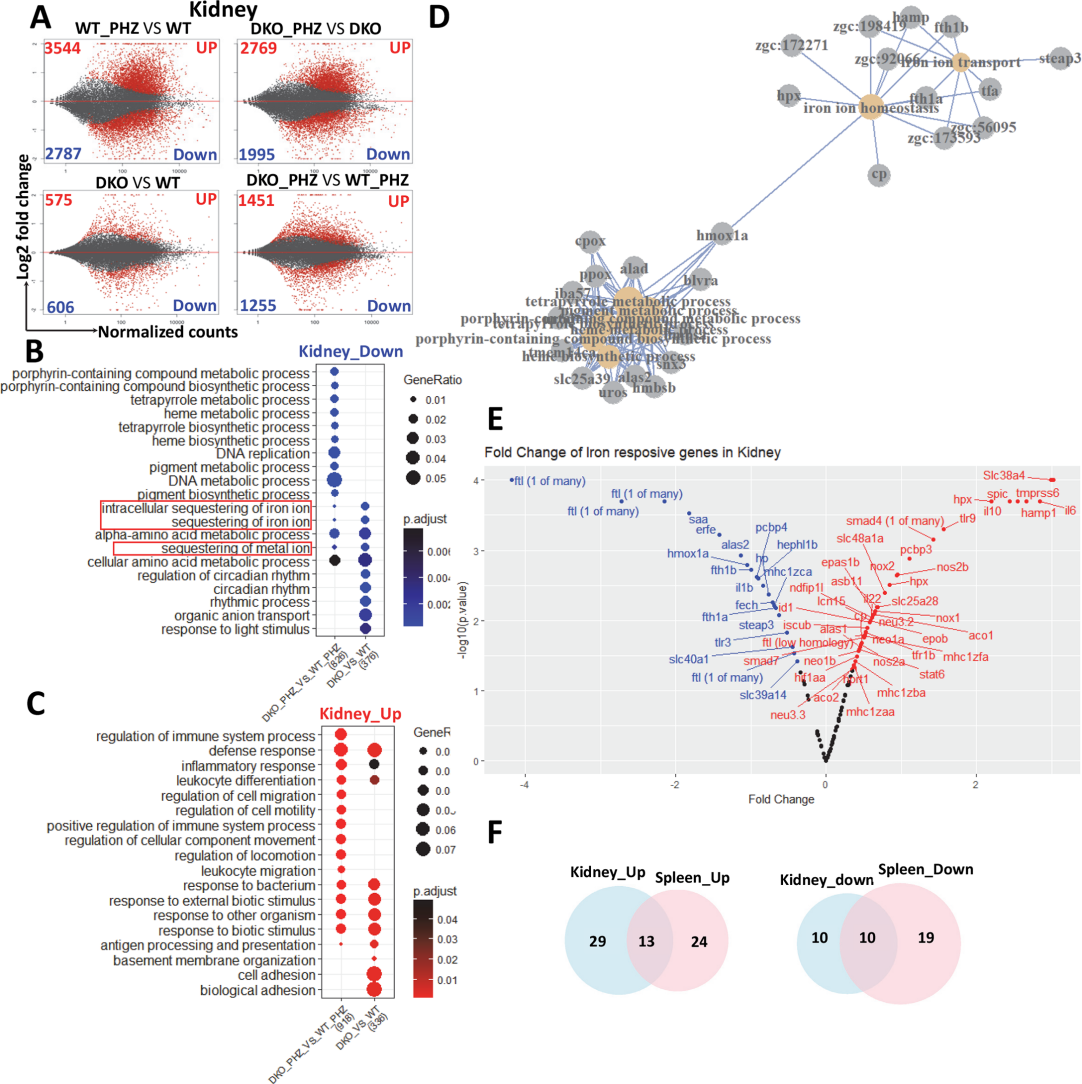
Genes related to heme-iron recycling are extensively perturbed in *hrg1* mutants. **(A)** MA plot of differentially expressed genes identified in kidney with pairwise comparison of WT_PHZ vs WT, DKO_PHZ vs DKO, DKO vs WT, DKO_PHZ vs WT_PHZ. Data represents individual gene expression alternation plotted as log_2_ fold-change versus baseMean normalized counts, with black and red dots representing non-significant and significant gene expression (p<0.05). Negative change representing the down-regulated genes and a positive change representing the up-regulated genes. **(B-C)** Enrichment analysis of GO biological processes related to differentially regulated genes in kidneys for comparison of DKO_PHZ vs WT_PHZ and DKO vs WT. Top 10 GO terms were shown together with common terms between PHZ and non-PHZ groups. The size of circles represents gene ratio to total annotated dataset, while p-values are indicated by color range (blue for down-regulated genes, and red for up-regulated genes). **(D)** Network association of GO biological processes and genes related to heme-iron metabolism which are down regulated in PHZ-treated DKO zebrafish comparing to PHZ-treated WT. **(E)** Fold Change of zebrafish homologues of iron-responsive gene between DKO and WT after PHZ treatment in kidneys. **(F)** Venn diagram showing numbers of common up- and down-regulated zebrafish homologues of iron-responsive gene between kidneys and spleens.

To determine whether heme and iron-dependent gene expression profiles were perturbed in *hrg1* mutant fish, we performed an RNA-seq on total RNA extracted from dissected kidneys and spleens of WT and *DKO* fish with or without PHZ-treatment. MA-plots of all 24,220 genes annotated in zebrafish genome GRCz10 [21] showed large numbers of differentially expressed genes in kidney and spleen when pairwise comparisons were performed between DKO and WT samples (**Fig 5A** and **S5A Fig**). Gene ontology (GO) enrichment analysis between PHZ-treated versus untreated kidney samples revealed that genes related to porphyrin metabolism, heme metabolism, and intracellular sequestering of iron were significantly downregulated, and immune-related genes were upregulated in the DKO kidney after PHZ treatment (**Fig 5B and 5C**). Strikingly, enrichment analysis to identify connecting pathways and interactomes from the GO data identified *hmox1a* connecting the heme/porphyrin metabolism sub-network with iron homeostasis (**Fig 5D**). On contrast to the kidney, GO analysis of the spleen data showed genes related to blood regulation and hemostasis were downregulated and cell-division genes were upregulated (**S5B and S5C Fig**).

To classify iron metabolism genes in zebrafish, we compiled a list of 86 mammalian iron metabolism genes and performed BLAST homology to identify 124 potential orthologs in the zebrafish genome (**S1 and S2 Table**). Further examination of these genes identified 20 genes that were significantly downregulated (*p<0*.*0001–0*.*038*) in the DKO kidneys after PHZ treatment (**Fig 5E**). These genes included ones involved in heme degradation (*hmox1a*), iron-storage and transport (*fth*, *ftl*, *slc40a1/fpn1*), heme synthesis (*alas2*, *fech*), and systemic iron regulation (*erfe*) (**Fig 5E**). By contrast, 42 genes involved in iron metabolism regulation (*hamp1*, *tmprss6*), macrophage differentiation (*spic*), and inflammation (*il6*, *il10*, *il22*) were upregulated (*p<0*.*0001–0*.*049*) (**Fig 5E**). Comparison of the iron and heme metabolism genes from the kidney and spleen revealed 10 downregulated and 13 upregulated genes that were common to both datasets (**Fig 5F, S5C and S5D Fig**). These results demonstrate that *hrg1* deficiency causes significant alterations in gene expression of heme and iron metabolism pathways in zebrafish.

## Discussion

In this study, we demonstrate the *in vivo* function of Hrg1 in a vertebrate model system. We show that the zebrafish kidney is the primary organ for heme-iron recycling during EP and that zebrafish Hrg1a and Hrg1b are heme transporters that are expressed and upregulated in kidney macrophages after PHZ-induced hemolysis. In agreement with this finding, genetic ablation of *hrg1* results in aberrant heme-iron metabolism at the histological and transcriptomic level.

In mammals, the spleen and liver are major organs for heme-iron recycling. In zebrafish, the kidney is the organ where adult hematopoiesis occurs, comparable to the bone marrow in mammals. However, a role in RBC turnover has not been ascribed to the zebrafish kidney. It has been postulated that the zebrafish spleen is the major organ for EP, despite a lack of direct experimental evidence [15, 16]. This rationale is supported by the observation that the majority of the zebrafish spleen is a reservoir of RBCs, with identifiable lymphatic or myeloid cells plus red and white pulps that are comparable to mammals [15, 16]. However, our studies show that the zebrafish kidney is the major site for EP after hemolysis. Indeed, iron staining reveals active heme-iron recycling in the zebrafish kidney, but not the spleen and liver even though expression of *hmox1a*, which encodes for the heme degrading enzyme, is upregulated in all three tissues [22].

We have previously used pre-mRNA splice-blocking morpholinos, which targeted the boundaries of *hrg1a* intron 2 and exon 3 (*hrg1a_I2E3_MO2*) and exon 2 and intron 2 (*hrg1a_E2I2_MO1*). Both morpholinos resulted in anemic embryos which could be partially rescued with expression of *hrg1*. Short sequence BLAST searches showed that the morpholino is specific to its targeting site with low off-target sequences in the zebrafish genome. How is it possible for the *hrg1a* morphants to show anemia while germline CRISPR mutants do not? It is possible that compensatory pathways can buffer against deleterious mutations, an effect typically not observed in transient morpholino knockdowns. Indeed, Rossi et al showed that *egfl7* mutants do not show any obvious phenotypes compared to morphants because Emilin2 and Emilin3 can compensate for loss of Egfl7 [23]. Another study showed that *tmem88a*^-/-^ mutant embryos partially recapitulated but had a much milder phenotype compared to *tmem88a* morphants [24]. One way to recapitulate the morphant phenotype would be to delete the morpholino target site by removing intron 2. Although we attempted to delete the entire *hrg1a and hrg1b* locus using two CRISPR guide RNAs, only F_0_ chimeras were recovered with no germline transmission in the F_1_ progeny even after screening large numbers (>300) of F_1_ embryos. We speculate that the *hrg1a* locus may harbor genetic elements which might be essential for embryonic development.

In humans, heme-iron recycling from senescent RBCs contributes to more than 90% of daily iron requirement, while dietary iron accounts for the remaining 10% [2, 3]. In zebrafish, the individual contribution of iron/heme absorption versus recycling is poorly understood. The zebrafish is typically fed a diet of brine shrimp and dry food extracts every day that is likely to be iron-loaded, together with iron absorbed from surrounding water. While regulation of iron absorption by the Fpn1-Hamp1 axis is well-documented in mice, it is noteworthy that zebrafish can absorb iron by an alternate pathway via their gills [25]. Indeed, we measured *hamp1* and *fpn1* mRNA by qRT-PCR in isolated livers from zebrafish. Our results show that *hamp1* levels are significantly elevated in the presence of PHZ but is equivalent in both, WT and DKO fish (**S6 Fig**). The upregulation of *hamp1* in response to PHZ is consistent with published studies in zebrafish embryos [26]. However, changes in *fpn1* expression is statistically not significant even though there is a trend of lower *fpn1* levels in DKO fish exposed to PHZ. Whether the Fpn1-Hamp1 axis regulates iron absorption from the gills is currently unknown. Therefore, one possible explanation for why the *hrg1* mutants lack overt erythropoietic phenotypes could be because zebrafish can obtain sufficient amounts of iron from dietary absorption or from the water [27].

Our RNA-seq results indicate that not only genes involved in heme-iron metabolism, but also immune-related genes are differentially expressed in PHZ-treated DKO zebrafish. One top hit of upregulated genes in DKO mutants is an unannotated gene named *si*:*ch211-201o1*.*1*, which is an NLRP3 homolog in zebrafish. It has been reported that an increase in extracellular heme activates NLRP3 expression, triggering inflammasome activation [28]. One possible explanation for this upregulation would be that the heme accumulating in the kidney macrophages in the absence of Hrg1 triggers an inflammatory response. It will be interesting to determine the immune response of Hrg1 mutant fish in the presence of pathogens and inflammatory agents that causes hemolysis.

Little is understood about red cell recycling and turnover in the adult zebrafish as the vast majority of studies related to red cell development and heme and iron metabolism have been confined to embryos. One major advantage of the zebrafish over other vertebrate models is the *ex utero* transparency of early-stage embryos which are easier to genetically and chemically manipulate. It is therefore noteworthy that *hrg1a* and *hrg1b* mRNA are both expressed in the one-cell embryo raising the possibility that Hrg1 and heme may play a hitherto underappreciated role in early embryonic development. Although there are no obvious phenotypes in the *DKO* mutant embryos, it is possible that these embryos may be more sensitive to maternal iron deficiency or disruption in heme biosynthesis. Here, generating transgenic zebrafish expressing genetically-encoded heme sensors/reporters [29, 30] or label-free imaging [31] to directly visualize heme trafficking at the maternal-embryonic interface in the transparent embryo, and within the hematopoietic organs during heme recycling in the adult fish will significantly influence our understanding of the role of heme and iron in vertebrate red cell development.

## Materials and methods

### Zebrafish husbandry and genotyping

All zebrafish procedures were approved by University of Maryland College Park Animal Care and Use Committee (#R-NOV-17-52). The Zebrafish Tü strain was used as wild-type. The light providing cycle was maintained at 10 hr light off and 14 hr light on. Embryos were kept in embryo medium prepared following *The zebrafish book* (4th). To keep embryos transparent in early developmental stages, 0.003% PTU was added around 18-24hpf.

For genotyping of adult zebrafish, a small piece of tail was clipped and placed in 50μl 50mM NaOH at 95°C for 30min. For genotyping of embryos, either whole embryos or a small piece of tail was dissolved in 10μl 50mM NaOH at 95°C for 30 min [32]. The lysates were neutralized by adding 1/10 volume of 1M Tris-HCl pH 8.0 and 1 μl of crude lysate was used for PCR genotyping. The PCR fragment of *hrg1a*^*iq261*^ and *hrg1b*^*iq361*^ alleles were genotyped with the following primers: for *hrg1a*, forward: 5’- GAATTATCAAGCTTCACATCACAGGCTCTTTCCGAG -3’, reverse: 5’- AAGCTACACTGCAGCACCGCTGTCTCCAGGTCAAACG -3’; for *hrg1b*, forward: 5’- actgcataGGATCCCCCTTTAAAGTGTGTTATCATGTG -3’, reverse: 5’- GCagactcctcgagCTTCCTACTACAGGGCCTGAATC -3’.

### PHZ treatment

For PHZ treatment in adult fish, 2.5 μg/ml PHZ was prepared in system fish water. Adult fish was placed in fish water with PHZ for 25 min at 28°C. PHZ was then rinsed off with fresh fish water.

### Histological section

Adult zebrafish (8–12 months old) were anesthetized with 0.02% tricaine (MS-222, Western Chemical Inc.) in fish water. Adult fish were first fixed by 10% Neutral buffered formalin (NBF) after slitting along the ventral abdominal wall to allow the fixative to immerse the gastrointestinal organs. The volume of fixative was at least 10 times of fish volume. The fixed fish were either processed as whole-mount paraffin embedded sections or frozen sections embedded in OCT after dissecting the kidneys, spleens and livers.

### Iron staining

Perl’s Prussian blue stain was performed to detect ferric iron zebrafish sections [33]. To perform DAB-enhanced staining, endogenous peroxidase activity was quenched by incubating embryos in 0.3% H_2_O_2_ (in methanol) for 20 min at RT. Following 3 times rinse in PBS, sections were incubated in DAB substrate kit (Pierce, Thermo Fisher) for 15 min. Ferric ferrocyanide catalyzes oxidation of DAB, producing a reddish-brown color.

### Whole-amount in situ hybridization

Whole amount in situ hybridization (WISH) was performed following standard protocol as described [34]. To generate probes for *hrg1a* and *hrg1b*, 3’UTR regions were amplified using following primers: *hrg1a* probe: forward, 5’- gcagtcacctcgagACACACAGCAGCACACTAGTGTC -3’, reverse, 5’- gatctaggatccGTCTGAGCGCAGCTGACAGAC -3’; *hrg1b* probe: forward, 5’- gcagactcctcgagTTGGCTCCTTCAGCTCTAATGG -3’, reverse, 5’- gatctcggatccGACTTAAACTGTATATTATTTCC -3’. The amplified fragments were cloned to pCS2+ vector with BamH1 and Xho1 digestion. Probes were synthesized by using DIG RNA Labeling Kit (SP6/T7) (Roche).

### Zebrafish tissue dissection

Dissection of various adult zebrafish tissue was performed as previously described [35]. For qRT-PCR experiments, dissected tissue was immediately placed in TRIzol and flash-frozen.

### Generation of zebrafish mutants by CRISPR/cas9 genome editing

The CRISPR gRNA was designed using Optimized CRISPR Design (http://crispr.mit.edu/). The gRNA target sequences for *hrg1a* (NM_200006.1) and *hrg1b* (NM_001002424.2) are listed: *hrg1a* exon 2: GGTGGATCTGACGACAGGAA TGG; *hrg1b* exon 3: GGCGGTAGCGGTAGGAGTAC AGG. The gRNA constructs were cloned using pT7-gRNA as backbone [36]. pCS2-Cas9 was used to produce Cas9 capped mRNA by *in vitro* transcription. Approximately ~300ng Cas9 mRNA and ~100ng gRNA were co-injected to embryos at 1-cell stage. Injected embryos were raised to adulthood as F0 chimeric founders. The founders were subjected to tail-clip genotyping to confirm indels at target sites. The positive chimeric founders were them crossed to WT zebrafish. The F1 embryos with indels at target sites were raised as stable mutant lines.

### RT-PCR

One microgram of total RNA was used for reverse transcription by iScript cDNA synthesis kit (Bio-Rad). The reaction without reverse transcriptase was used for a negative control. cDNA was diluted 2–5 times for following PCR reaction.qRT-PCR was performed with SsoAdvanced Universal SYBR Green Supermix (Bio-Rad). Each reaction was triplicated to avoid possible random variations.

### *O-*dianisidine staining

*O-*dianisidine staining was performed to detect hemoglobin in RBCs of whole embryos or histological sections as previously described [37]. Heme catalyzes oxidation of *o-*dianisidine in the presence of H_2_O_2_, producing a dark brown color in hemoglobin-positive cells. Briefly, collected embryos or sections were placed in 1 ml staining solution (0.06 (w/v) *O-*dianisidine, 25% Ethanol, 10mM Sodium Acetate and 0.02% H_2_O_2_) for 20 min in dark conditions. Staining was stopped by rinsing with 70% ethanol.

### FACS cell sorting for cell quantification

FACS analysis was performed as described [38]. Embryos from globinLCR: GFP transgenic background were pooled. The cells were disaggregated and then sequentially filtered through 70 μm and 33 μm cell strainers. The percentage of GFP-positive cells in transgenic embryos was analyzed by FACSCantos II machine (BD Biosciences).

### Membrane fractionation

Dechlorinated zebrafish embryos or dissected adult tissues were disrupted using a Dounce Homogenizer in appropriate volume of homogenization buffer (10mM Tris-HCl, mM EDTA, 1mM PMSF, 1X protease inhibitor cocktail (Roche)). The homogenized solution was then centrifuged at 800 g at 4°C for 5 min. The supernatant was ultra-centrifuged at 100, 000 g at 4°C for 90 min. The supernatant is the cytosolic fractionation and the pellet was treated as crude membrane fraction. The pellet was collected and dissolved in lysis buffer (2% Triton-X100, 150mM NaCl, 50mM Tris-HCl, 20mM HEPES, 1mM PMSF, 1mM EDTA, 1X protease inhibitor cocktail). The membrane lysate was used Western blot experiments.

### Immunoblotting

Polyclonal HRG1 antibody serum was generated in rabbit using the C-terminal 17 amino acid peptide sequence (YAHRYRADFADIILSDF) of human Hrg1 as antigen (Epitomics, Inc.). Since the C-terminal 17 amino acid sequence of human Hrg1 has high homology to zebrafish Hrg1a and Hrg1b (15/17), it cross-reacts with both Hrg1a and Hrg1b.

For western blot analysis of Hrg1 protein in zebrafish, total protein concentration in membrane fractionation lysate was measured using the Pierce BCA assay kit (Thermo Scientific). Equal amount of total protein was mixed with Laemmli sample buffer and were separated on 12% SDS-PAGE and transferred to a 45 μM nitrocellulose membrane with semi-dry transfer apparatus (Bio-Rad). The affinity purified Hrg1 antibody was used at a concentration of 1:1000, goat anti-rabbit HRP-conjugated secondary was used at 1: 30,000, and blots were developed in SuperWest Femto Chemiluminescent Substrate (Thermo Scientific).

### Collection of red blood cells

Embryos were anesthetized in of 0.02% tricaine in embryos medium, 1% bovine serum albumin (BSA) in calcium- and magnesium-free PBS. The tails of approximately 30 embryos were cut with surgical scissors to allow red blood cells (RBCs) to flow into the tricaine solution. The tricaine solution containing RBCs was loaded into Shandon EZ Single Cytofunnels (Thermal scientific) and concentrated onto a slide (Thermal scientific) by centrifugation at 450 rpm for 3 mins using a Shandon Cytospin 4 cytocentrifuge (Thermal scientific) according to the manufactory’s instructions. Slides were air-dried prior to May-Grunwald Giemsa staining.

### May-Grünwald giemsa staining

May-Grünwald staining solution (May-Grünwald solution (MG500, sigma-aldrich): methanol = 1:3) was gently added onto slides, incubated 5 min at RT and the stain rinsed off with distilled water. Subsequently, the slides were incubated with 1 ml Giemsa staining solution (Giemsa Stain (GS500, Sigma-Aldrich): water = 1:20) for 15 to 30 mins. The Giemsa staining solution was then washed off with distilled water and slides were air-dried before examining under microscope.

### Yeast growth essay and western blotting

The *S*. *cerevisiae* strain W303 containing the *hem1Δ* mutation has been described previously [39, 40]. The mutant yeast cells were maintained at 30°C in yeast peptone dextrose (YPD) media supplemented with 250 μM δ-aminolevulinic acid (ALA) (Frontier Scientific). To generate yeast expression plasmids, the zebrafish *hrg1a* and *hrg1b* ORFs were cloned with primers containing BamHI and XbaI restriction sites into the pYES-DEST52 vector (Invitrogen). The mutant alleles of *hrg1a* and *hrg1b* were amplified with primers containing BamH1 and XbaI sites (with and without a C-terminal HA tag), and cloned into pYES-DEST52.

The dilution spot assays were performed as described previously [39, 40]. Plasmids containing potential heme transporters were transformed into *hem1Δ* yeast using the lithium method [41]. To assay aerobic growth exclusively, yeast was induced with 2% galactose in the of ALA, and then spotted onto plates containing indicated heme or ALA concentrations as well as 2% glycerol and 2% lactate as a carbon source. The western blotting experiments were performed as previously reported [19].

### RNA-seq and data analysis

The kidneys from 5–6 month old adult zebrafish (Tü, *DKO*, non-PHZ and PHZ-treated) were dissected and flash-frozen in TRIzol before RNA extraction (To minimize variations from individual fish, tissues from three adult zebrafish were pooled for each of the three biological replicates. 3 fish as a cohort, 3 cohorts per genotype). Total RNA was extracted following TRIzol manual (Invitrogen). The extracted RNA was digested with RNase free-DNase to remove remaining genomic DNA and cleaned up using Qiagen RNA mini column (Qiagen, Germany). Quality and quantity of total RNA were checked by Agilent Bioanalyzer 2100. One microgram of total kidney RNA and 100 ng of total spleen RNA were used for RNA-seq library construction. Purified mRNA was prepared from total RNA following the manufactory’s manual of NEBNext Poly-A) mRNA Magnetic Isolation Module (E7490S, New England Biolabs). RNA-seq libraries was constructed with NEBNext Ultra RNA Library Prep Kit for Illumina (E7530L, New England Biolabs). The RNA-seq libraries and fragment size was roughly qualified by Agilent Bioanalyzer 2100. RNA-seq libraries were quantified using NEBNext Library Quant Kit for Illumina (E7630S, New England Biolabs).

RNA-seq was performed using Illumina’s HiSeq-2500. Total of 24 samples with single-end 50 base reads were sequenced, with triplicate libraries of spleens and kidneys. Bioinformatics quality control was done using FastQC, version 0.11.5. The reads were aligned to zebrafish GRCz10reference genome using STAR, version 2.5.2b. The numbers of reads mapped to genes were counted using HTSEQ, version 0.6.1p1. Finally, differentially expressed genes were identified via DESeq2, version 1.12.3 with the cutoff of 0.05 on False Discovery Rate (FDR). R version 3.3.2 (2016-10-31) was used, and Bioconductor version 3.4 with BioInstaller version 1.24.0 were used. For gene annotation, we used Ensembl GRCz10, release 87. False Discovery Rate (FDR) by Benjamini-Hochberg was used to determine the statistical significance with the cutoff value of 0.05. GO enrichment and network analysis were performed using R package clusterProfiler [42]. All the sequencing data including read counts per gene were deposited to GEO with the accession number of GSE109978.

## Supporting information

S1 Fig Expression of *hrg1a* and *hrg1b* in embryonic and adult zebrafish. **(A-B)** WISH of *hrg1a* and *hrg1b* expression in embryos at different stages. Anterior is to the left. Anti-sense probe is used to detect mRNA expression; sense probe is shown to indicate background staining. Scale bar: 200μm. **(C-D)** qRT-PCR on *hrg1a* and *hrg1b* in dissected adult zebrafish tissues from male and female. 3 male or female fish were dissected as one cohort, each gender had 3 cohorts as biological replicates. Expression level was normalized to *ef1α*.
(TIF)

S2 Fig *DKO* double knockout mutants can survive to adulthood without overt hematological phenotypes. **(A)** Schematic structure of *hrg1a* and *hrg1b* mutant alleles with genomic sequences at the CRISPR/Cas9 targeting sites. For *hrg1a*, the targeting site is on exon 2, mutant allele *hrg1a*^*iq261*^ carries a mutation with 61nt deletion and 7nt (TGGATCT) insertion, causing the deletion of the ATG (red font) translation start site. For Hrg1b, the targeting sequencing is on exon 3, mutant allele *hrg1b*^*i361*^ has a 61nt deletion. Targeting sequences are showing as underscored characters. PAMs (Protospacer adjacent motif) are showing as *italic f*ont. **(B)** Western blot showing mutant forms of Hrg1a and Hrg1b are expressed in yeast. Hrg1a^iq261^ is generated by using downstream alternative ATG translation start site, with deletion of 38aa at N-terminus. Epitope-tagged Hrg1b^iq361^ was used for yeast growth assay, and HA western blot showed both WT *hrg1b* and *hrg1b*^*iq361*^ were expressed in yeast. However, expression level of Hrg1b^iq361^-HA was low. **(C)** Genotyping of *hrg1a*^*iq261/iq261*^*; hrg1b* ^*iq361/iq361*^, and *DKO*. *Hrg1a*^*iq261/iq261*^ has an indel of -61nt, +7nt in exon 2 and *hrg1b* ^*iq361/iq361*^ carries -61nt deletion in exon 3, causing small-sized PCR products. **(D)** Genotyping of progenies from intercross of *hrg1a*^*+/iq261*^*; hrg1b*^*+/iq361*^ at the stage of 3dpf with expected Mendelian ratio. No significant difference (Chi-square test, p>0.05). **(E)** Representative FACS plot to show percentages of GFP^+^ cells in globinLCR-GFP embryos. **(F)** *O-*dianisidine staining showing hemoglobinization of isolated RBCs from 3dpf embryos peripheral blood. **(G)** *O-*dianisidine staining showing hemoglobinization of isolated RBCs from adult peripheral blood.
(TIF)

S3 Fig GFP IHC for zebrafish sections. **(A)** GFP IHC of liver sections from adult transgenic zebrafish *gata1*:*gfp* and *mpeg1*:*gfp* with control (non-PHZ) and 1 day post PHZ-treatment. **(B)** GFP IHC of kidney and liver sections from Tü WT zebrafish with control (non-PHZ) and 1 day post PHZ-treatment.
(TIF)

S4 Fig *hrg1* DKO zebrafish undergoes normal erythrophagocytosis with defects in heme-iron recycling. **(A)** Perl’s Prussian blue iron staining of kidney sections from Tü and *hrg1* DKO zebrafish with control (non-PHZ), 1 day, 2 days and 3 days post PHZ-treatment. Yellow arrows: macrophages. **(B)** DAB-enhanced perl’s iron staining of spleen sections from Tü and *hrg1* DKO zebrafish with control (non-PHZ), 1 day, 2 days and 3 days post PHZ-treatment. **(C)** IHC staining of Hrg1 proteins in kidney, spleen and liver of adult *hrg1* DKO zebrafish sections. **(D)** H&E staining of kidney, spleen and liver sections from *hrg1* DKO zebrafish with control (non-PHZ) and 1-day post PHZ-treatment. Scale bar: 20μm.
(TIF)

S5 Fig Whole transcriptome analysis of differentially expressed genes in *hrg1* DKO mutants. **(A)** MA plot of differentially expressed genes identified in spleens with pairwise comparison of WT_PHZ vs WT, DKO_PHZ vs DKO, DKO vs WT, DKO_PHZ vs WT_PHZ. Data represents individual gene expression alternation plotted as log_2_ fold-change versus baseMean normalized counts, with black and red dots representing non-significant and significant gene expression (p<0.05). Negative change representing the down-regulated genes and a positive change representing the up-regulated genes. **(B-C)** Enrichment analysis of GO biological processes related to significantly down- and up-regulated genes in spleens for comparison of DKO_PHZ vs WT_PHZ and DKO vs WT. **(D)** Fold Change of zebrafish homologues of iron-responsive gene between DKO and WT after PHZ treatment in spleens.
(TIF)

S6 Fig Expression of *fpn1* and *hamp1* in the liver of WT and DKO adult zebrafish. **(A)** qRT-PCR of *fpn1* mRNA expression in the liver from control (non-PHZ) and PHZ treated adult zebrafish at one-day post treatment. **(B)** qRT-PCR of *hamp1* mRNA expression in the liver from control (non-PHZ) and PHZ treated adult zebrafish at one-day post treatment. **** p < 0.0001.
(TIF)

S1 Table Fold changes of iron-responsive genes in the zebrafish kidney. (XLSX)

S2 Table Fold changes of iron-responsive genes in the zebrafish spleen. (XLSX)
