# Supplementary figures and images for: Hrg1 promotes heme-iron recycling during hemolysis in the zebrafish kidney
Source: PLoS Genet. 2018 Sep 24;14(9):e1007665. doi: 10.1371/journal.pgen.1007665 (PMC6171960; doi:10.1371/journal.pgen.1007665)

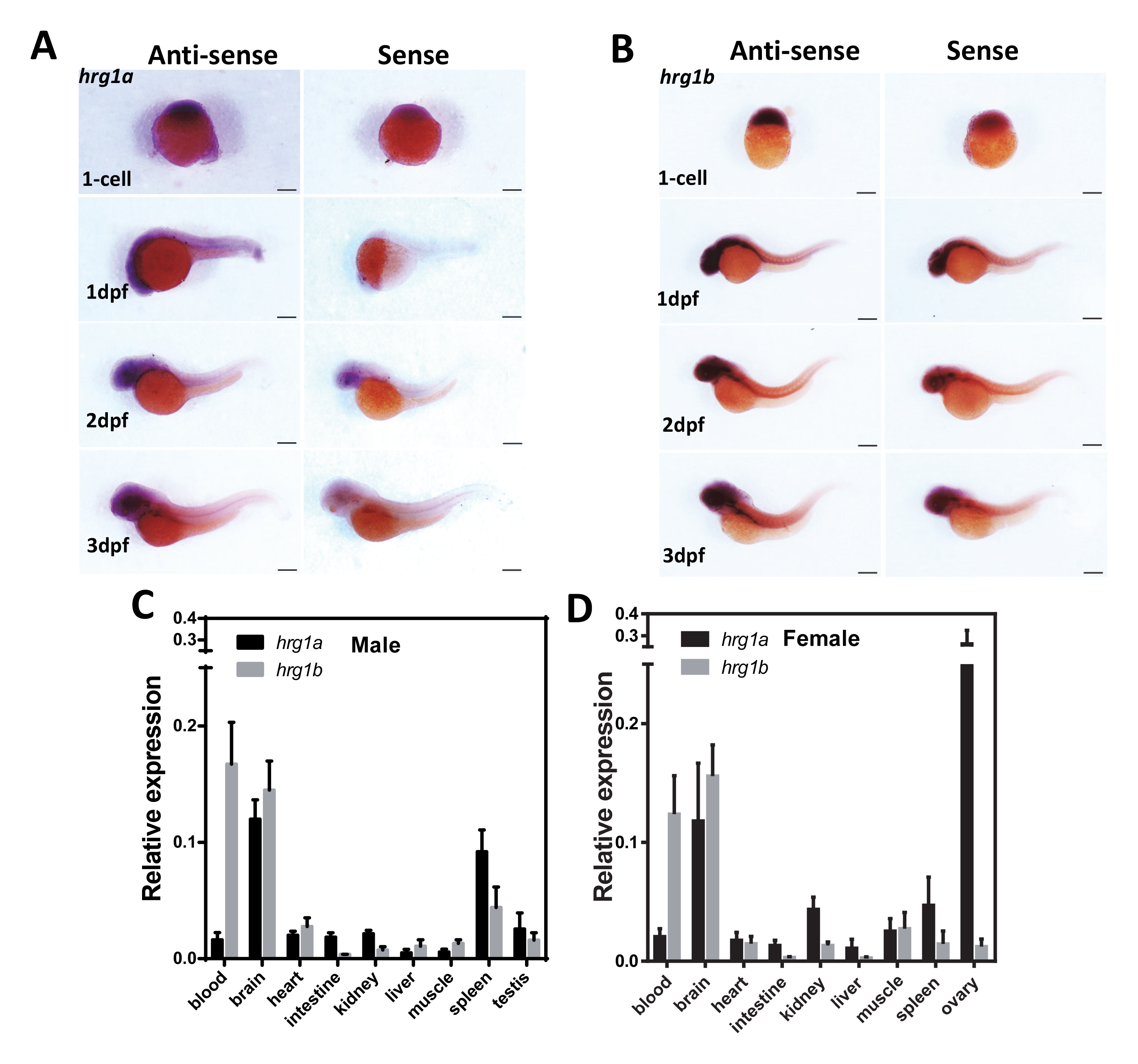

Supplement: S1 Fig — (A-B) WISH of hrg1a and hrg1b expression in embryos at different stages. Anterior is to the left. Anti-sense probe is used to detect mRNA expression; sense probe is shown to indicate background staining. Scale bar: 200μm. (C-D) qRT-PCR on hrg1a and hrg1b in dissected adult zebrafish tissues from male and female. 3 male or female fish were dissected as one cohort, each gender had 3 cohorts as biological replicates. Expression level was normalized to ef1α. (TIF) [file pgen.1007665.s001.tif]

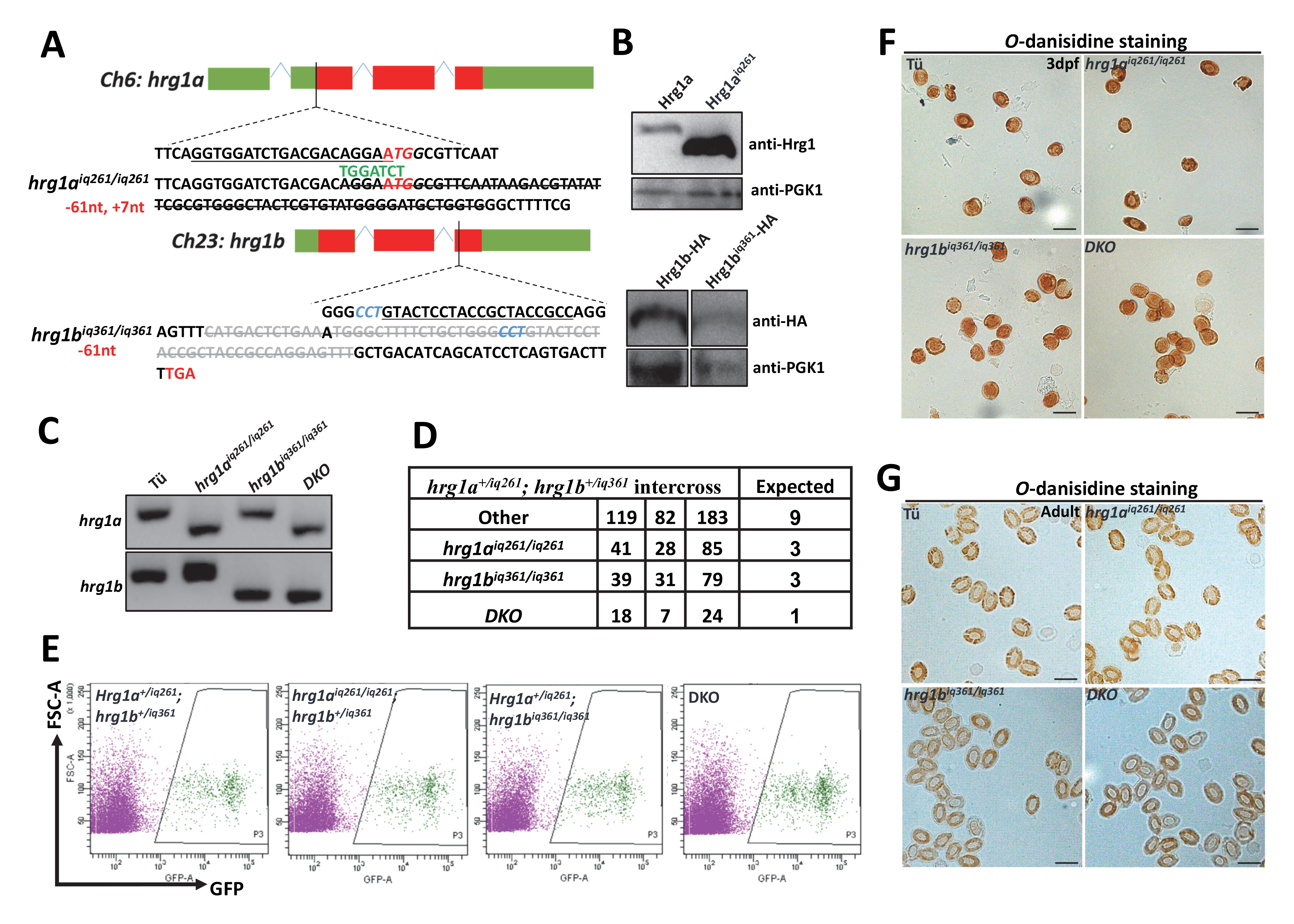

Supplement: S2 Fig — (A) Schematic structure of hrg1a and hrg1b mutant alleles with genomic sequences at the CRISPR/Cas9 targeting sites. For hrg1a, the targeting site is on exon 2, mutant allele hrg1aiq261 carries a mutation with 61nt deletion and 7nt (TGGATCT) insertion, causing the deletion of the ATG (red font) translation start site. For Hrg1b, the targeting sequencing is on exon 3, mutant allele hrg1bi361 has a 61nt deletion. Targeting sequences are showing as underscored characters. PAMs (Protospacer adjacent motif) are showing as italic font. (B) Western blot showing mutant forms of Hrg1a and Hrg1b are expressed in yeast. Hrg1aiq261 is generated by using downstream alternative ATG translation start site, with deletion of 38aa at N-terminus. Epitope-tagged Hrg1biq361 was used for yeast growth assay, and HA western blot showed both WT hrg1b and hrg1biq361 were expressed in yeast. However, expression level of Hrg1biq361-HA was low. (C) Genotyping of hrg1aiq261/iq261; hrg1b iq361/iq361, and DKO. Hrg1aiq261/iq261 has an indel of -61nt, +7nt in exon 2 and hrg1b iq361/iq361 carries -61nt deletion in exon 3, causing small-sized PCR products. (D) Genotyping of progenies from intercross of hrg1a+/iq261; hrg1b+/iq361 at the stage of 3dpf with expected Mendelian ratio. No significant difference (Chi-square test, p>0.05). (E) Representative FACS plot to show percentages of GFP+ cells in globinLCR-GFP embryos. (F) O-dianisidine staining showing hemoglobinization of isolated RBCs from 3dpf embryos peripheral blood. (G) O-dianisidine staining showing hemoglobinization of isolated RBCs from adult peripheral blood. (TIF) [file pgen.1007665.s002.tif]

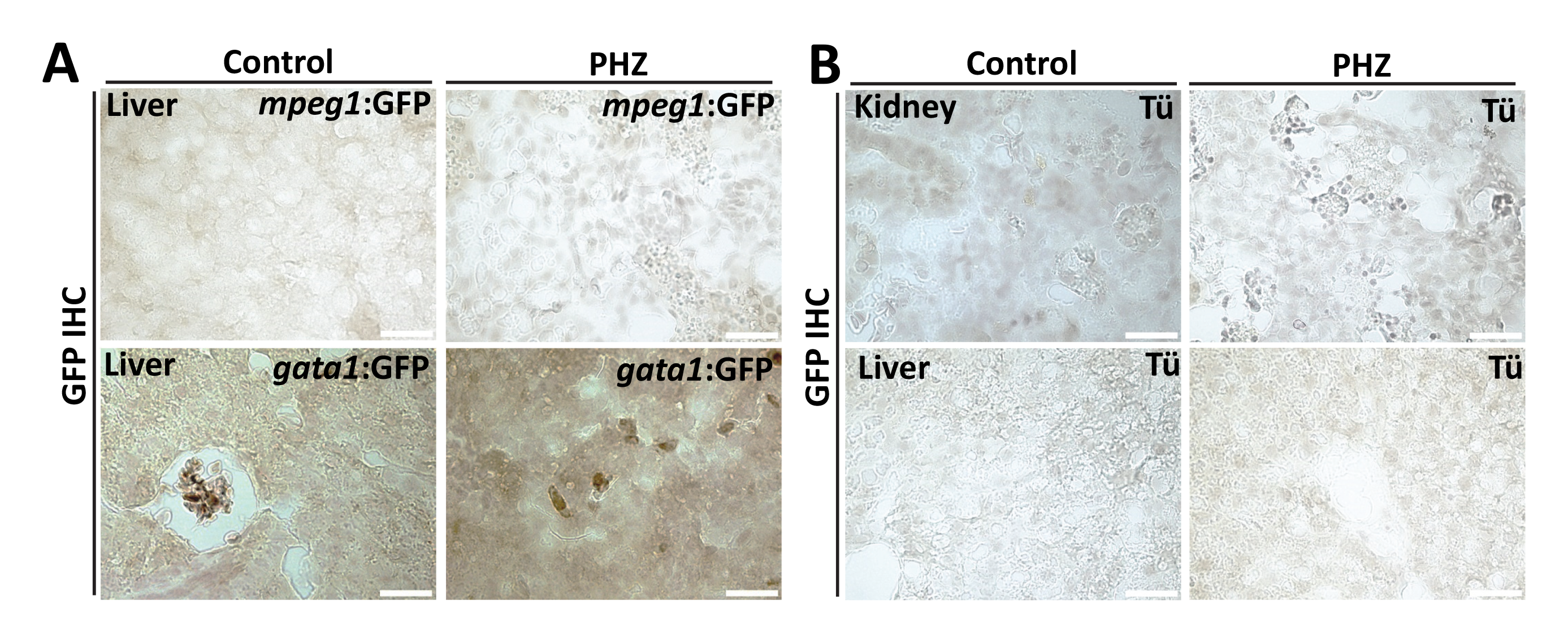

Supplement: S3 Fig — (A) GFP IHC of liver sections from adult transgenic zebrafish gata1:gfp and mpeg1:gfp with control (non-PHZ) and 1 day post PHZ-treatment. (B) GFP IHC of kidney and liver sections from Tü WT zebrafish with control (non-PHZ) and 1 day post PHZ-treatment. (TIF) [file pgen.1007665.s003.tif]

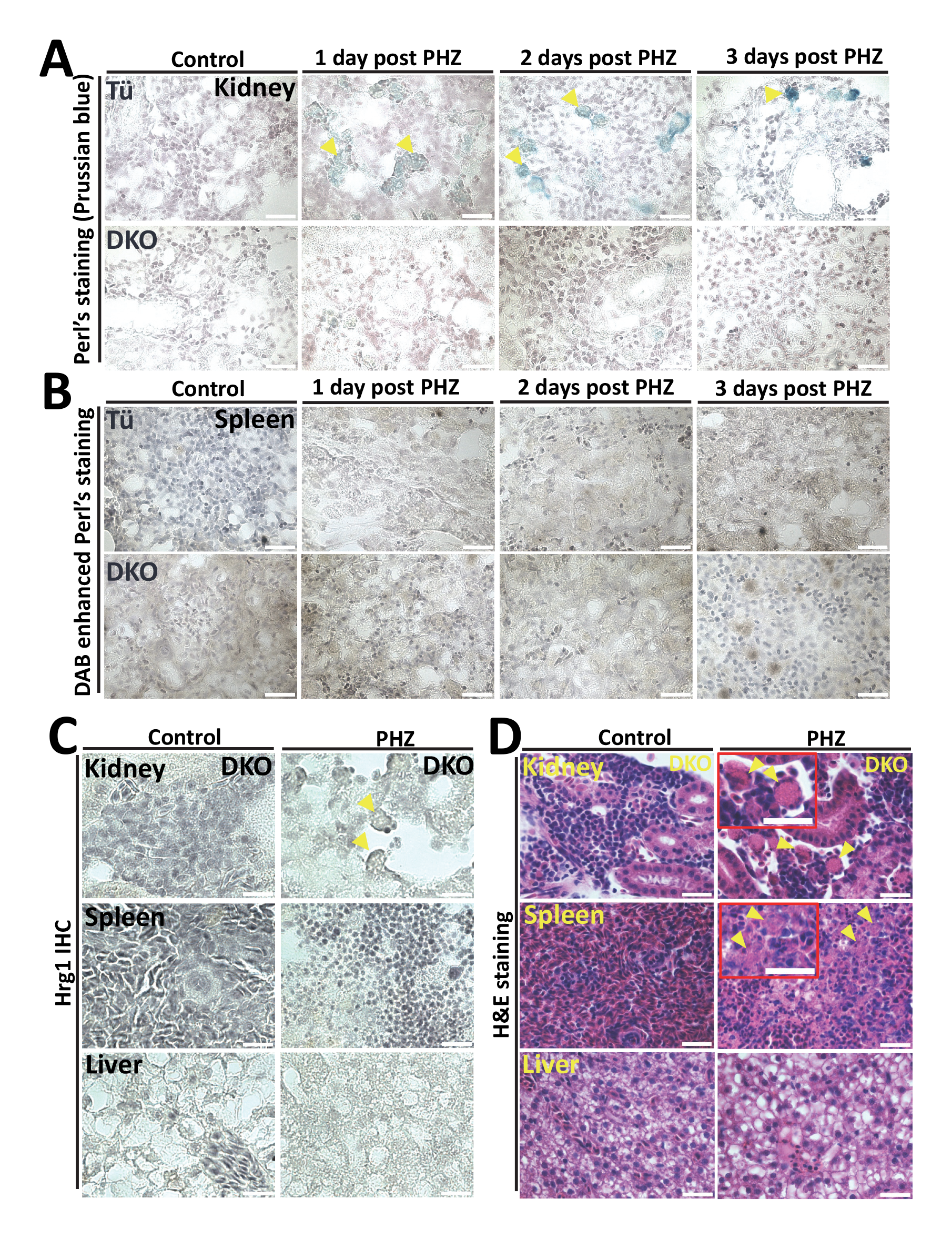

Supplement: S4 Fig — (A) Perl’s Prussian blue iron staining of kidney sections from Tü and hrg1 DKO zebrafish with control (non-PHZ), 1 day, 2 days and 3 days post PHZ-treatment. Yellow arrows: macrophages. (B) DAB-enhanced perl’s iron staining of spleen sections from Tü and hrg1 DKO zebrafish with control (non-PHZ), 1 day, 2 days and 3 days post PHZ-treatment. (C) IHC staining of Hrg1 proteins in kidney, spleen and liver of adult hrg1 DKO zebrafish sections. (D) H&E staining of kidney, spleen and liver sections from hrg1 DKO zebrafish with control (non-PHZ) and 1-day post PHZ-treatment. Scale bar: 20μm. (TIF) [file pgen.1007665.s004.tif]

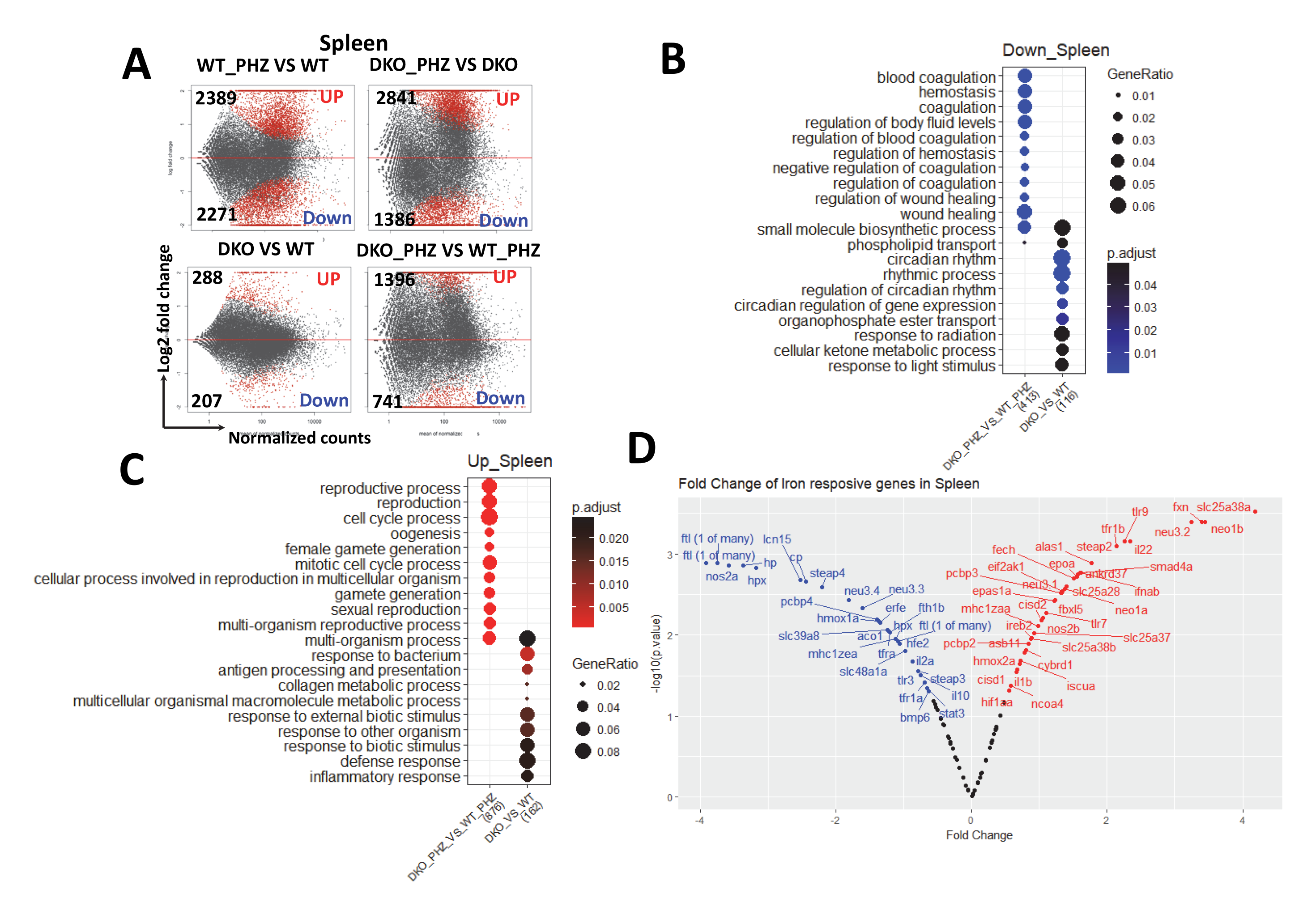

Supplement: S5 Fig — (A) MA plot of differentially expressed genes identified in spleens with pairwise comparison of WT_PHZ vs WT, DKO_PHZ vs DKO, DKO vs WT, DKO_PHZ vs WT_PHZ. Data represents individual gene expression alternation plotted as log2 fold-change versus baseMean normalized counts, with black and red dots representing non-significant and significant gene expression (p<0.05). Negative change representing the down-regulated genes and a positive change representing the up-regulated genes. (B-C) Enrichment analysis of GO biological processes related to significantly down- and up-regulated genes in spleens for comparison of DKO_PHZ vs WT_PHZ and DKO vs WT. (D) Fold Change of zebrafish homologues of iron-responsive gene between DKO and WT after PHZ treatment in spleens. (TIF) [file pgen.1007665.s005.tif]

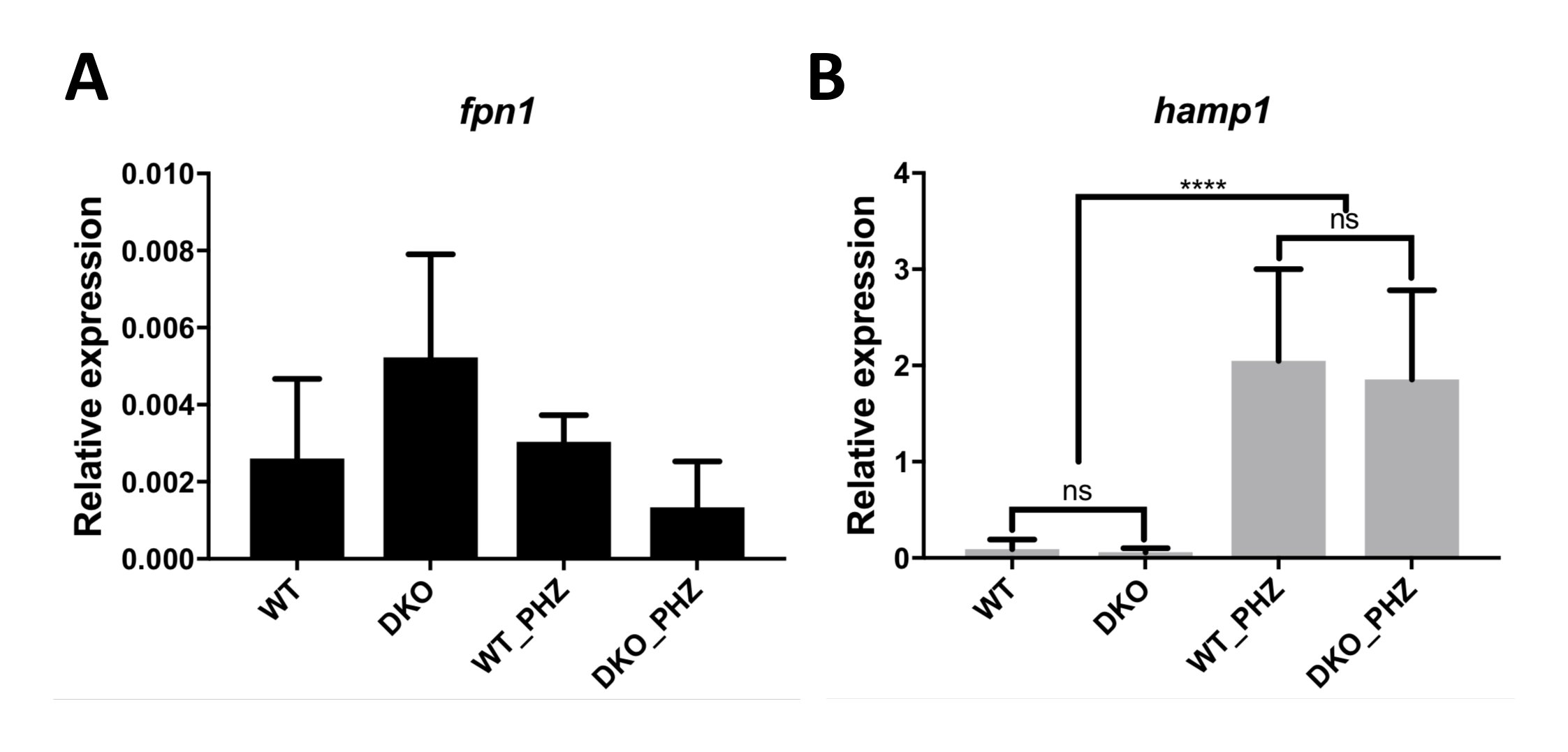

Supplement: S6 Fig — (A) qRT-PCR of fpn1 mRNA expression in the liver from control (non-PHZ) and PHZ treated adult zebrafish at one-day post treatment. (B) qRT-PCR of hamp1 mRNA expression in the liver from control (non-PHZ) and PHZ treated adult zebrafish at one-day post treatment. **** p < 0.0001. (TIF) [file pgen.1007665.s006.tif]
